# Supplementary material for: Bacteroides thetaiotaomicron enhances H2S production in Bilophila wadsworthia
Source: Gut Microbes. 2024 Nov 28;16(1):2431644. doi: 10.1080/19490976.2024.2431644 (PMC11610557; doi:10.1080/19490976.2024.2431644)
Supplement: Supplemental Material [file KGMI_A_2431644_SM8187.zip › Supplementary Figures.docx]

#
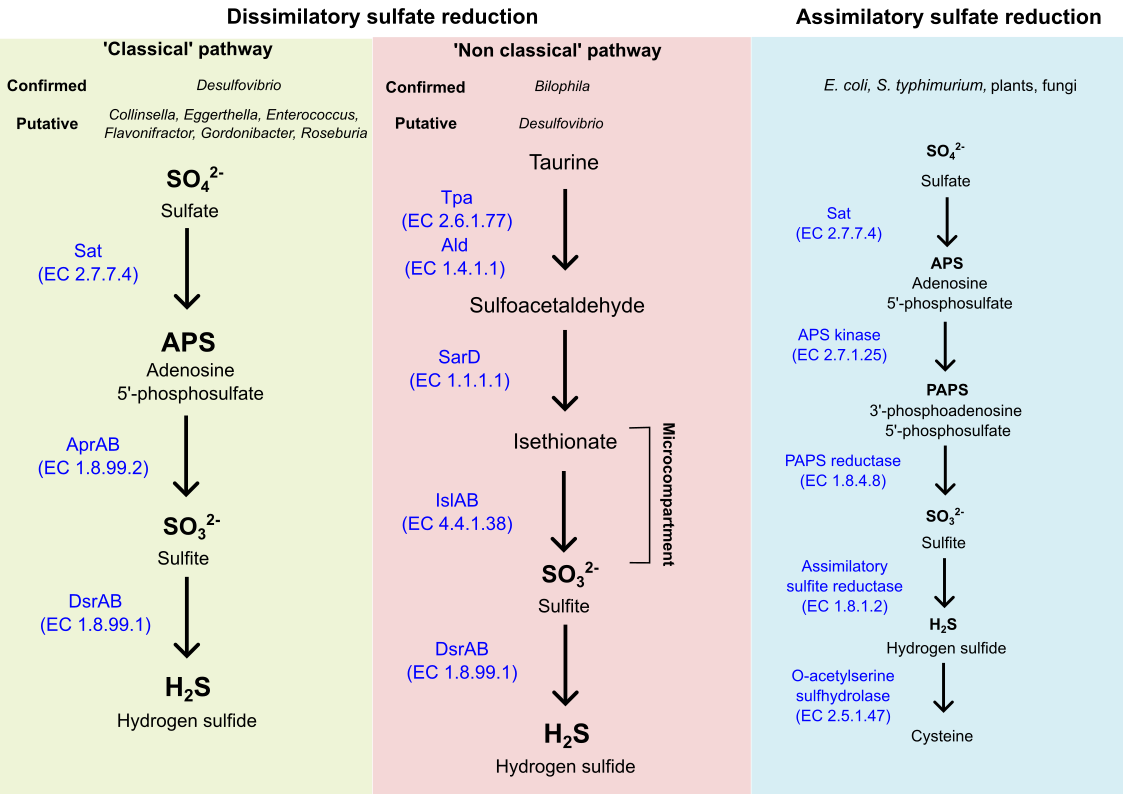
Supplementary Figures

**Figure S1: Metabolic pathways for sulfate reduction** Dissimilatory sulfate reduction is a strictly anaerobic process performed by sulfate-reducing bacteria (SRB). Toxic isethionate intermediates are encapsulated within microcompartments in *B. wadsworthia* *^1^*.


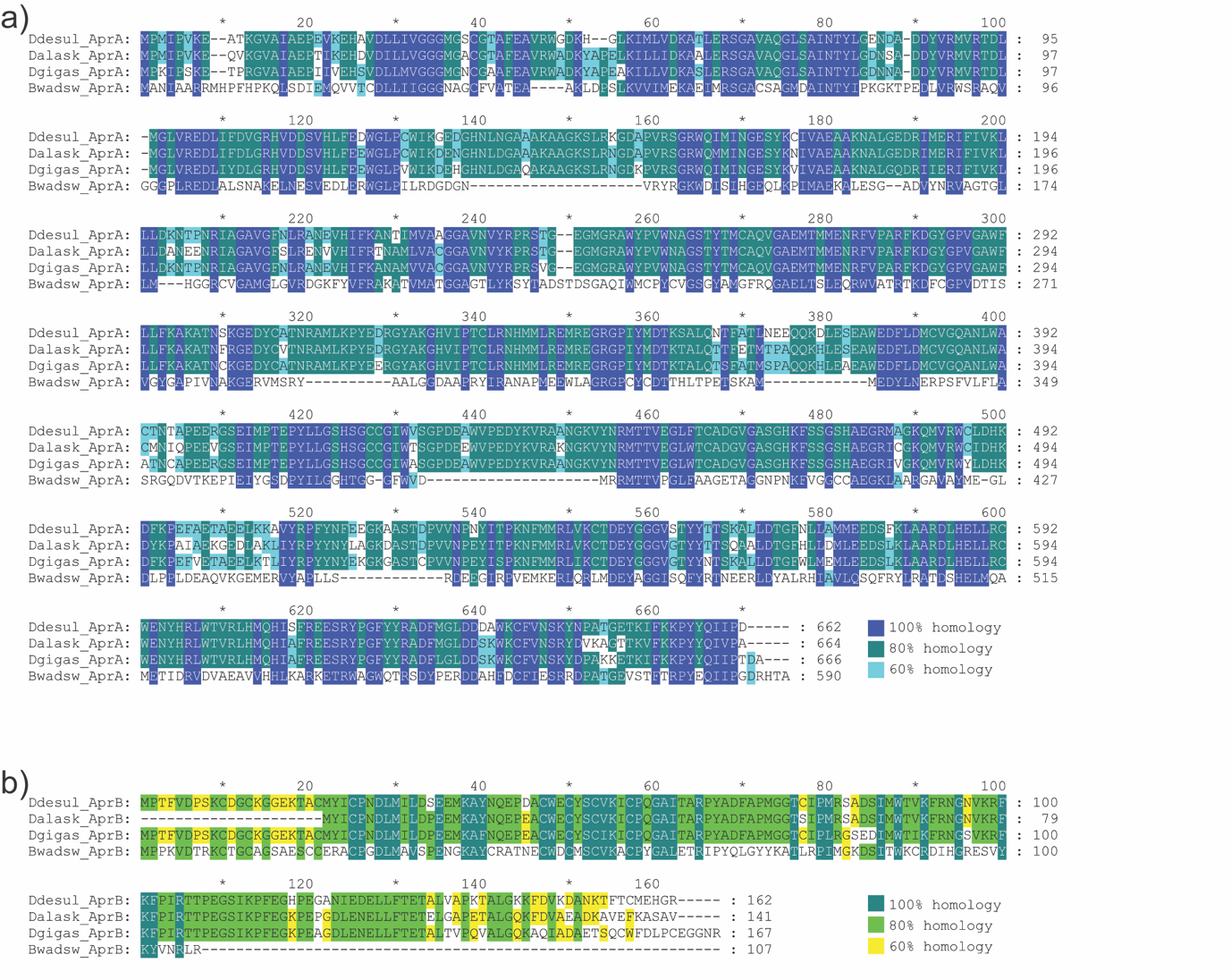


**Figure S2:** Alignment of amino acid sequences of **a)** AprA genes and **b)** AprB genes from B. wadsworthia QI0013 (Bwadsw), Desulfovibrio gigas DSM 1382 (Dgigas), Desulfovibrio alaskensis G20 (Dalask) and Desulfovibrio desulfuricans subsp. desulfuricans DSM 642 (Ddesul).

| 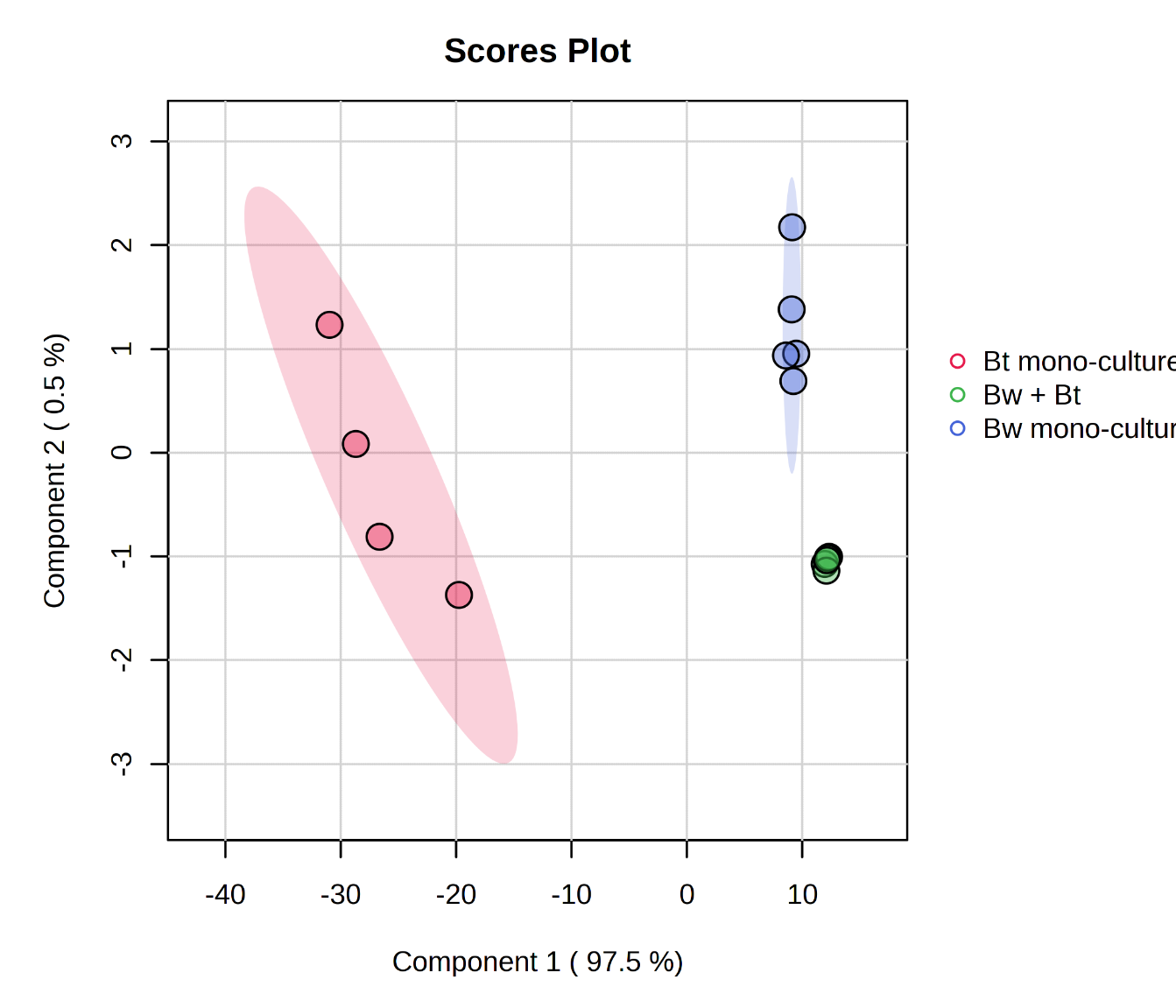  **a)**  Bt monoculture  Bw + Bt  Bw monoculture |
| --- |
| 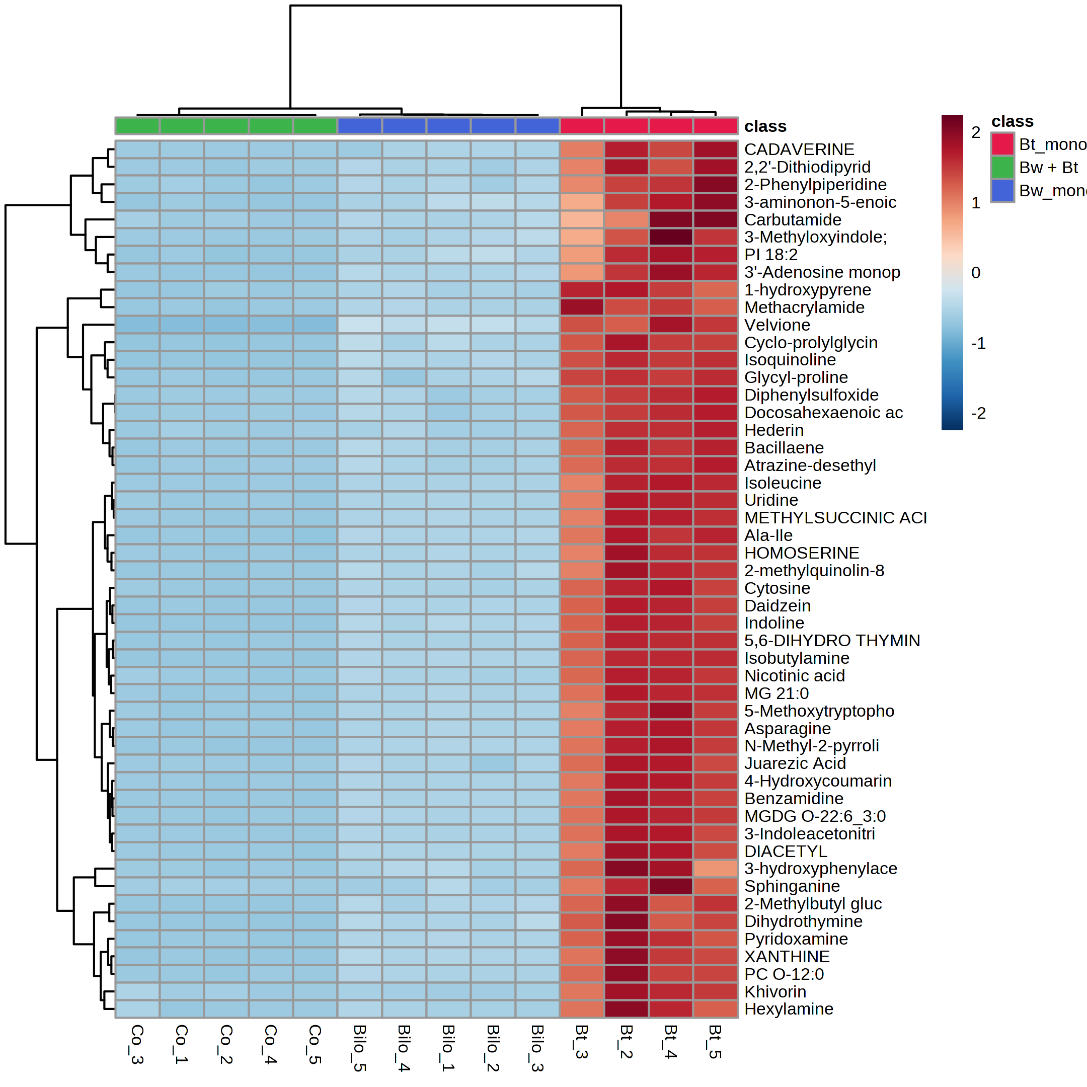  **b)**  Bt mono 4  Bt mono 3  Bt mono 1  Bt mono 2  Bw mono 3  Bw mono 2  Bw mono 1  Bw mono 4  Bw mono 5  Bw + Bt 5  Bw + Bt 4  Bw + Bt 2  Bw + Bt 1  Bw + Bt 3  Bt monoculture  Bw + Bt  Bw monoculture  Cadaverine  2,2'-Dithiodipyridine  2-Phenylpiperidine  3-aminonon-5-enoic acid  Carbutamide  3-methyloxyindole  PI 18:2  3'-adenosine monophosphate  1-hydroxypyrene  Methacrylamide  Velvione  Cyclo-prolylglycine  Isoquinoline  Glycyl-proline  Diphenylsulfoxide  Docosahexaenoic acid  Hederin  Bacillanene  Atrazine-desethyl  Isoleucine  Uridine  Methylsuccinic acid  Ala-Ile  Homoserine  2-methylquinolin-8-ol  Cytosine  Daidzein  Indoline  5,6-dihydro thymine  Isobutylamine  Nicotinic acid  MG 21:0  5-Methoxytryptophol  Asparagine  N-methyl-2-pyrrolidone  Juarezic acid  4-hydroxycoumarin  Benzamidine  MGDG O-22:6_3:0  3-Indoleacetonitrile  Diacetyl  3-hydroxyphenylacetic acid  Sphinganine  2-Methylbutyl glucopyranoside  Dihydrothymine  Pyridoxamine  Xanthine  PC O-12:0  Khivorin  Hexylamine |

**Figure S3:** Comparisons of the endometabolome of *B. wadsworthia* and *B. thetaiotaomicron* in co-culture (Bw + Bt) with monocultures (Bw_mono, Bt_mono). **a)** PLS-DA plot of metabolites acquired from samples via untargeted LC-MS in positive ion mode. **b)** Heatmap displaying relative abundance of top 50 differentially abundant metabolites in the culture conditions.
