# Supplementary material for: Bacteroides thetaiotaomicron enhances H2S production in Bilophila wadsworthia
Source: Gut Microbes. 2024 Nov 28;16(1):2431644. doi: 10.1080/19490976.2024.2431644 (PMC11610557; doi:10.1080/19490976.2024.2431644)
Supplement: Supplemental Material [file KGMI_A_2431644_SM8187.zip › FiguresLegends.docx]

# Figure legends

**Figure 1: Co-culture of *B. wadsworthia* (Bw) QI0013 and *B. thetaiotaomicron* (Bt). a)** H_2_S concentrations (µM) at 8 h of *B. wadsworthia* in monoculture or in co-culture with *B. thetaiotaomicron* QI0072, DSM 108160, and DSM 108161. **b)** H_2_S concentration (µM) per 10^6^ *B. wadsworthia* cells at 8 h. **c-d)** Cell counts of *B. wadsworthia* and *B. thetaiotaomicron* at 8 h post-inoculation measured via qPCR. Each point represents a culture replicate (n=7). In the negative control bacterial cells were not added. **e-f)** The cell numbers were tracked over time for the co-culture of *B wadsworthia* QI0013 and *B. thetaiotaomicron* QI0072. Horizontal lines represent average, and error bars represent SD. Results of unpaired t-tests are shown where ** = p ≤ 0.01, *** = p ≤ 0.001, **** = p ≤ 0.0001.

**Figure 2: Differentially expressed genes (DEGs) of *B. wadsworthia* and *B. thetaiotaomicron* in co-culture versus monoculture.** a) DEGs in *B. wadsworthia*. b) DEGs in *B. thetaiotaomicron*. Bar charts show numbers of DEGs increased (green) and decreased (red) in expression in co-culture relative to the respective monoculture in each functional gene class as annotated in BV-BRC.

**Figure 3: Model of cross-feeding interaction between *B. wadsworthia* and *B. thetaiotaomicron*.** Enzymes are shown in colours corresponding to transcriptomic data: green showed increased gene expression in co-culture; red showed decreased expression in co-culture; grey showed no change in expression between co-culture and monoculture. The blue star represents a putative enzyme where functionality is unproven. The dotted line represents a putative cross-feeding mechanism. Dashed lines represent electron transfer.

**Figure 4: Growth (OD_600_) of *B. wadsworthia* in ABB media supplemented with 10 mM adenosine 5’-phosphosulfate (APS), 4 mM sulfite or 10 mM taurine.** Growth was compared to *B. wadsworthia* grown in ABB media. Each box plot represents the median and interquartile range of the distribution of 7 culture replicate. Results of mixed linear model analyses are shown where * = p ≤ 0.01, ** = p ≤ 0.001.

**Figure 5: Pairwise co-culture of *B.* thetaiotaomicron (Bt) QI0072 with four *B. wadsworthia* (Bw) strains (QI0012, QI0013, QI0014, QI0015).** a) H_2_S concentrations (µM) at 8 h. **b)** qPCR-determined *B. wadsworthia* cell counts at 8 h. **c)** H_2_S concentration (µM) per 10^6^ *B. wadsworthia* cells. **d)** qPCR-determined *B. thetaiotaomicron* cell counts at 8 h. Each point represents a culture replicate (n=3). Horizontal lines represent average, and error bars represent SD. Statistical significance between culture conditions was established using one-way analysis of variance (ANOVA) with Tukey's multiple comparison tests with a significance level set at α = 0.05. Results show * = p ≤ 0.05, ** = p ≤ 0.01, *** = p ≤ 0.001, **** = p ≤ 0.0001, ns = not significant (p > 0.05).

**Figure 6:** Comparisons of the endometabolome of *B. wadsworthia* and *B. thetaiotaomicron* in co-culture (Bw + Bt) with monocultures (Bw_mono, Bt_mono). a) The top compounds ranked based on the Variable Importance in Projection (VIP) scores. The coloured boxes on the right indicate the relative concentrations of the corresponding metabolite in each group. b) Specific metabolites of interest manually curated from the compound list detected via untargeted LC-MS. c) Relative intracellular concentrations of compounds related to tryptophan metabolism in *B. thetaiotaomicron* (Bt) in monoculture and co-culture with *B. wadsworthia* (Bw + Bt). Concentration is standardised to *B. thetaiotaomicron* cell counts. Each point represents a culture replicate. Box and whisker plots show line at mean, box at 25^th^ – 75^th^ percentile, whiskers to minimum and maximum.

# References:

1 Burrichter, A. G. *et al.* Bacterial microcompartments for isethionate desulfonation in the taurine-degrading human-gut bacterium *Bilophila wadsworthia*. *BMC Microbiol.* **21**, 340 (2021). <https://doi.org:10.1186/s12866-021-02386-w>
